# Proliferation makes a substantive contribution to the maintenance of airway resident memory T-cell subsets in young pigs

**DOI:** 10.1093/discim/kyaf007

**Published:** 2025-04-12

**Authors:** Eleni Vatzia, Yan Zhang, Ehsan Sedaghat-Rostami, Veronica Martini, Basudev Paudyal, Brigid Veronica Carr, Adam McNee, Chris Chiu, Katy Moffat, Becca Asquith, Peter Beverley, Derek Macallan, Elma Tchilian

**Affiliations:** The Pirbright Institute, Pirbright, UK; Institute for Infection and Immunity, City St George’s, University of London, London, UK; The Pirbright Institute, Pirbright, UK; Section of Immunology, School of Biosciences, Faculty of Health and Medical Sciences, University of Surrey, Guilford, UK; The Pirbright Institute, Pirbright, UK; The Pirbright Institute, Pirbright, UK; The Pirbright Institute, Pirbright, UK; The Pirbright Institute, Pirbright, UK; The Pirbright Institute, Pirbright, UK; The Pirbright Institute, Pirbright, UK; Department of Infectious Disease, Imperial College London, London, UK; Department of Infectious Disease, Imperial College London, London, UK; Institute for Infection and Immunity, City St George’s, University of London, London, UK; The Pirbright Institute, Pirbright, UK

**Keywords:** pig, T cells, cell turnover, lung tissue-resident memory cell, cell division

## Abstract

Tissue-resident memory (TRM) T cells play an important role in protection against respiratory infection but whether this memory is maintained by long-lived or dividing cells remains controversial. To address the rate of division of lung TRM T cells, deuterium-enriched water was administered orally to young pigs to label dividing lymphocytes. T-cell subsets were separated from blood, lymph nodes, and airways [bronchoalveolar lavage (BAL)], the latter comprising almost exclusively TRM. We show that, as in other species, circulating memory T-cell subsets divide more rapidly than naïve T cells. Rates of labelling of memory subsets were similar in blood and lymph nodes, consistent with the rapid and free exchange. Strikingly, the fraction of label in BAL was similar to those in blood/lymph nodes after 5–21 days of labelling, suggesting replacement with recently divided cells, but this was preceded at Day 2 by a phase when labelling was lower in BAL than blood/lymph node in some memory subsets. Our data exclude long-lived TRM as the source of BAL memory cells leaving three possible hypotheses: blood/airway exchange, *in situ* proliferation, or proliferation in the lung interstitium followed by migration to BAL. When considered in the context of other information, we favour the latter interpretation. These results indicate the dynamic nature of memory in the lung and have implications for harnessing immune responses against respiratory pathogens.

## Graphical Abstract

**Graphical Abstract:**
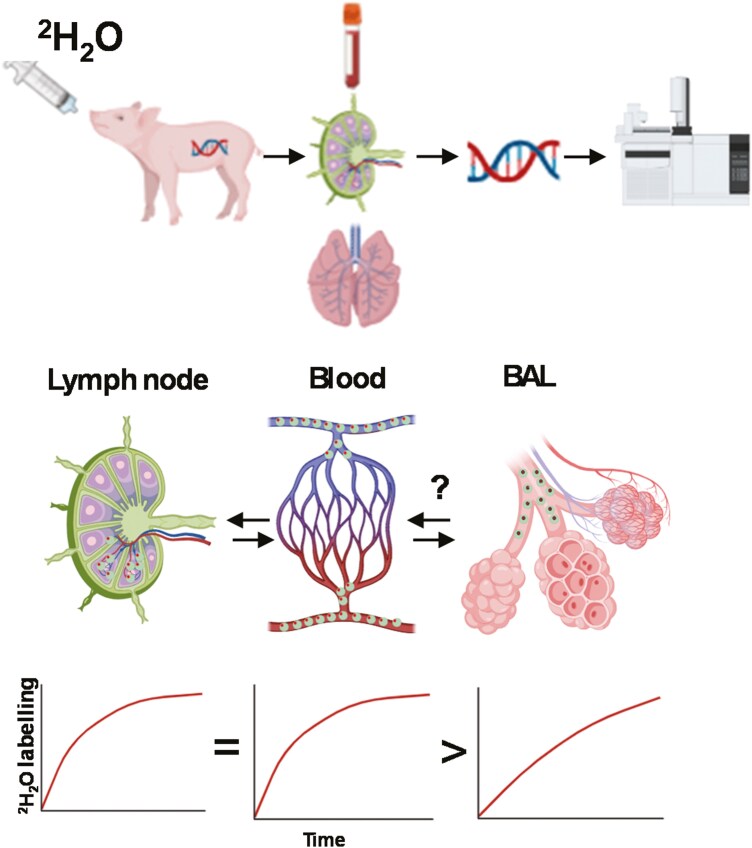

## Introduction

Understanding how memory is induced and maintained is of great importance for understanding immunity and for the design of more effective and long-lasting vaccines. Previously it was thought that memory predominantly resided in lymphocytes that circulated through blood and lymphoid organs but now it is clear that large numbers of memory lymphocytes are present in non-lymphoid tissues. Many of these are CD8 T cells found at mucosal sites where they are crucial for protection. The lung is one such site and lung tissue-resident memory (TRM) cells are pivotal in protection against respiratory viral infections [1, 2]. How they are generated and maintained, however, remains unclear, particularly in larger animals and humans.

For αβ T cells in general, following the first encounter with antigen, previously quiescent naïve T cells divide rapidly and differentiate to form clones of effector and memory cells. We and others have previously shown in humans that whilst naïve cells divide very infrequently, the majority of recirculating clonal progeny continue to divide and die throughout life with a relatively short lifespan [3, 4]. More recently, a smaller population of circulating stem cells like memory T cells, which may maintain memory over long periods of time, has also been identified [5, 6]. However, there is little consensus as to how memory cells in tissues such as the lung, are maintained once *in situ*—specifically whether this is due to cellular longevity or ongoing proliferation and replacement.

The persistence of TRM has been extensively studied in mice by tracking transgenic T cells or T cells with differing surface antigen alleles in parabiosis experiments. These experiments indicate that while there is rapid mixing of T cells in blood and lymphoid organs, this is not the case in non-lymphoid tissues, although there are differences between organs. There is little replacement of TRM in the skin, salivary glands, the reproductive tract, and small intestine over 200 days, but even in the lung, which has challenging microenvironmental features [7], only 40% of TRM are replaced over the same time period [8]. These mouse studies are in accord with human data indicating that TRM populations persist for long periods of time in grafted organs [9, 10].

In contrast to these data, which indicate that TRM populations persist for relatively long periods of time, there is much less information on how they persist *in situ*—specifically whether this results from cellular longevity or is due to ongoing proliferation, although there is some mouse and human data suggesting a slower rate of cell division of TRM compared to memory T cells in lymphoid tissues [11–13].

In this study, we sought to determine the rate of cell division of TRM. We chose to study the pig lung since the pig respiratory tract has many anatomical and physiological similarities to humans and the pig is a natural host for similar influenza A viruses to those found in humans [14–17]. The expanding toolkit for studying porcine immune responses, coupled with advances in annotating the porcine genome and the application of scRNA sequence analysis, has significantly enhanced the utility of the pig as a biomedical model [18, 19].

**Figure 1.**
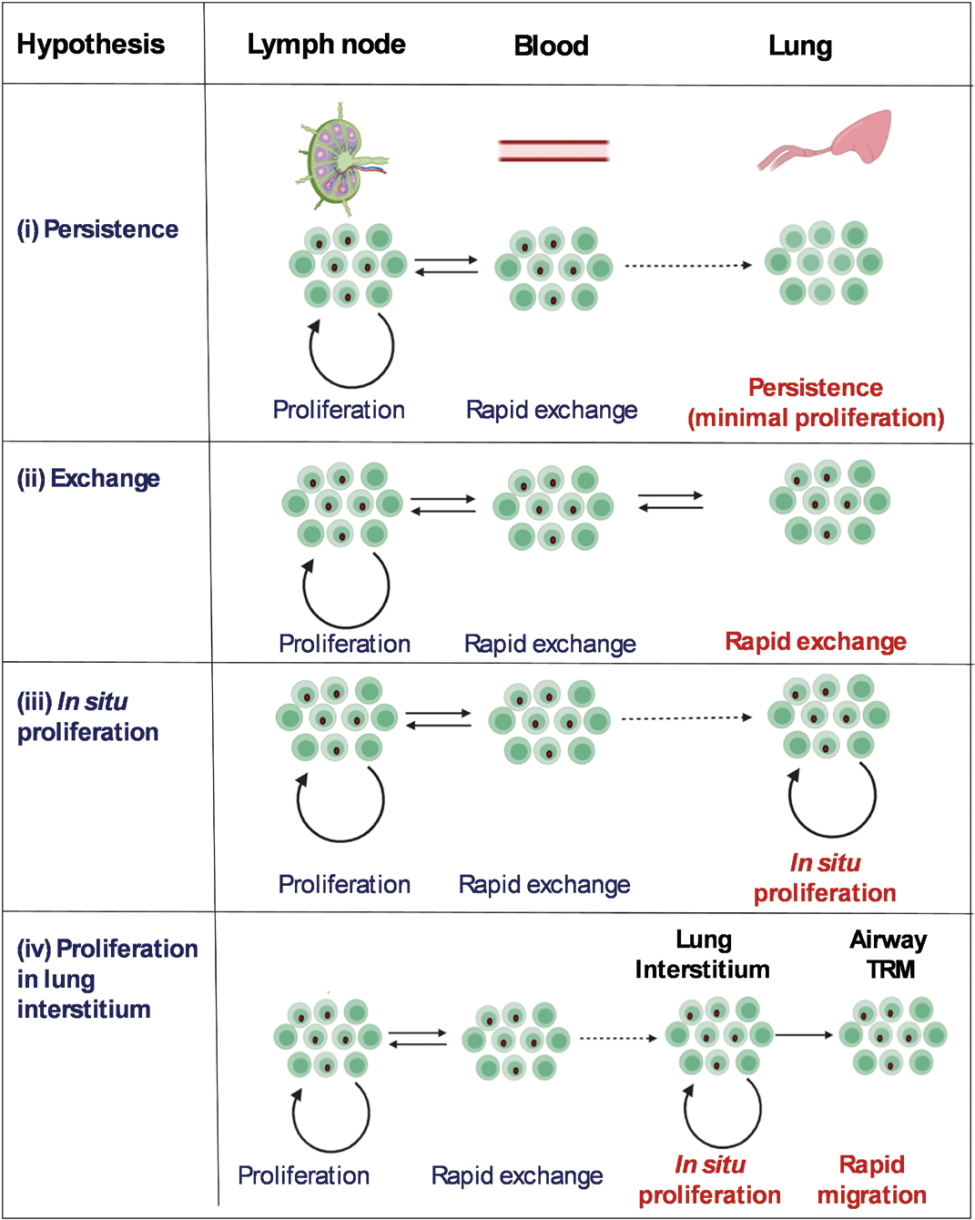
Four hypotheses for the maintenance of lung TRM in pigs. The four frames illustrate four potential hypotheses considered. Dots in cell nuclei represent deuterium-labelled DNA in recently divided cells—at high proportions for illustrative purposes. For simplicity, death *in situ* or efflux of cells from the airways is not shown in Models i, iii, and iv.

Because it is extremely difficult to extract and separate cleanly interstitial lung TRM from contaminating blood lymphocytes, we chose to study T cells present in bronchoalveolar lavage (BAL). These cells express markers characteristic of TRM and are considered extravascular as they are not labelled rapidly by intravenous CD3 antibodies [15, 20–22]. We hypothesize that the process of BAL recovers TRM present in the superficial layers of respiratory epithelium and airways. Because no similar studies had been performed in pig, it was first necessary to develop methods to label dividing T cells in pigs. Having done so, we asked whether TRM present in BAL shows evidence of recent division and, if so, how their division rates compare to memory T cells present in blood and lymph nodes. Specifically, we set out to test four hypotheses (Fig. 1): (i) that TRM populations persist because TRM cells are long-lived and do not divide *in situ*; (ii) that BAL TRM is maintained by proliferation in blood/lymph nodes with subsequent migration into the airway; (iii) that BAL TRM is maintained by proliferation *in situ*; or (iv) that BAL TRM is maintained by proliferation in the lung interstitium, followed by migration to BAL.

## Materials and methods

### Animal studies

**Figure 2.**
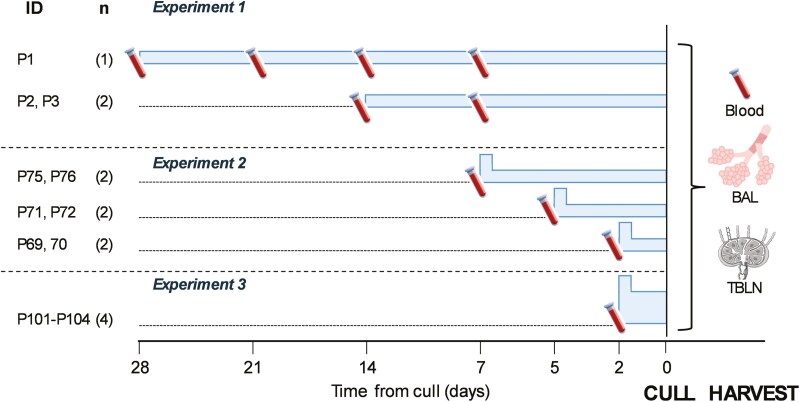
Study schematic. Study schematic showing interventions relative to the time of culling (Time 0) when blood, BAL, and TBLN were collected. Horizontal blue bars represent periods of heavy water labelling—the vertical height of the bar represents the relative magnitude of the dose (greater in Experiment 3); raised steps indicate priming doses. Blood tube icons indicate intermediate blood sampling times and tissue icons, post-mortem tissue sampling.

All experiments were approved by the ethical review processes at the Pirbright Institute and Animal and Plant Health Agency (APHA) and conducted according to the UK Government Animal (Scientific Procedures) Act 1986 supported by Project License P47CE0FF2. Animals were commercial-herd Landrace × Hampshire cross female pigs aged 5–7 weeks. They remained free-living within pens in a barn with *ad libitum* access to food and water and were acclimatized for at least 1 week prior to study procedures. Deuterium-enriched water (heavy water, 70% ^2^H_2_O, CK gases, Leicester, UK) was administered daily mixed with sweetened blackcurrant juice drink (Ribena, Suntory Beverage and Food GB&I)—the timing, dose, and duration of labelling varied by an animal (Fig. 2).

Three experiments were performed. In the first, three animals (P1–P3) received 35 ml of deuterium-enriched water for 14–28 days (Fig. 2). P3 also received a single dose of single cycle replication candidate influenza vaccine, S-Flu (kindly provided by Professor Alain Townsend, University of Oxford) by aerosol using a vibrating mesh nebulizer and face mask on the first day of labelling but the impact of vaccination *per se* is not addressed in this paper [15, 23]. Weekly blood samples were collected during labelling. At the end of the labelling period, animals were culled and BAL, tracheobronchial lymph node (TBLN), and blood samples were collected. Alignment of culling dates for logistic reasons meant that P2 and P3 started labelling two weeks after P1 and weighed more at label initiation (~18 kg versus P1, ~12 kg, Fig. 2 and Supplementary Fig. S1).

One objective of this study was to identify optimal labelling protocols for the porcine model. From Experiment 1 data it became apparent that many subpopulations had fully labelled by the time of culling, hence Experiment 2 was designed to capture labelling dynamics at earlier time points. We included a priming dose, 100 ml deuterium-enriched water on the first day of labelling, followed by 35 ml daily, as before, and culled animals in pairs on Days 2 (P69 and P70), 5 (P71 and 72), and 7 (P75 and P76) (Fig. 2). Blood, TBLN, and BAL were collected.

From Experiments 1 and 2 the importance of the early time-point data became apparent alongside an awareness that Day 2 labelling rates in cellular DNA were in the low range for measurement. Hence, in Experiment 3, we repeated the 2-day experiment with 4 pigs (P101–P104, the average weight of 11 kg) but with larger doses of deuterium-enriched water (2 × 180 ml on initiation day, then 1 × 90 ml the next day), culling at 48 hours post-initiation (Fig. 2).

### Sample collection and processing

BAL was collected from the entire lung after administration of 100 ml of PBS and gentle massage. BAL cells were isolated by centrifugation of the lavage fluid (500 g, 10 minutes). The cell pellet was washed in PBS, filtered through a 70 μM cell strainer, and cryopreserved. Heparinized blood samples were diluted 1:1 in PBS before density gradient centrifugation at 1200 × *g* for 30 minutes over Histopaque® 1.083 g/ml (Sigma). Peripheral blood mononuclear cells (PBMCs) were washed, red blood cells lysed with ammonium chloride lysis buffer, and cells cryopreserved. TBLNs were dissected out and dissociated into a single-cell suspension by mashing with the plunger of a syringe. The single-cell suspension was filtered twice using a 70 μM cell strainer, washed, and red blood cells lysed. Cells were washed again and cryopreserved. Plasma was also collected in some animals.

### Flow cytometry staining and fluorescence-activated cell sorting

PBMC, TBLN, and BAL cells were stained after depletion of monocytes/macrophages by magnetic bead depletion (MACS, Miltenyi) using CD172a-PE antibody (PE Mouse Anti-Pig Monocyte/Granulocyte, Clone 74-22-15A, BD) and Ultra-Pure PE beads (130-105-639, Miltenyi).

Unbound cells from the flow-through were surface labelled with a live/dead dye (LIVE/DEAD™ Fixable Near-IR Dead Cell Stain Kit, Thermo Fisher Scientific), CCR7-BV711 (CD197, Rat Anti-Human, Clone 3D12, BD), CD4-PerCP-Cy™ 5.5 (Mouse Anti-Pig CD4a, Clone 74-12-4, BD), CD45RA-FITC (Mouse Anti-Pig, Clone MIL13, Bio-Rad), CD8 beta-PE (Mouse Anti-Pig CD8 Beta, Clone PPT23, Bio-Rad), and CD3-APC (Mouse Anti-Porcine CD3e-APC, Clone 4510-11, Cambridge Bioscience). Cells were sorted using a FACSAria III (BD Biosciences) and frozen prior to analysis for DNA deuterium enrichment. Bound cells were retrieved from the column and designated ‘monocytes’ (from blood) and ‘alveolar macrophages’ (from BAL) for subsequent analysis; purity was confirmed by flow cytometry.

Using the gating strategy shown in Supplementary Fig. S2, we separated the blood and TBLN CD4^+^ T cells into CD45RA^+^CCR7^+^ (naïve, TN), CD45RA^−^CCR7^+^ (central memory, TCM), and CD45RA^−^CCR7^−^ (effector memory, TEM); and CD8 T cells into CD45RA^+^CCR7^+^ (TN), CD45RA^−^CCR7^+^ (TCM), CD45RA^−^CCR7^−^ (TEM), and CD45RA^+^CCR7^−^ (terminally differentiated effectors, TDE). BAL cells were separated into corresponding populations (if present) using the same markers. The enrichment of CD3-positive cells following monocyte/alveolar macrophage depletion using CD172 beads is shown in Supplementary Fig. S3.

Three pigs (X1, X2, and X3) from the same batch, which were not given deuterium (Pigs X1–X3), were infused intravenously with 2.4 mg/kg in 10 ml purified CD3 Ab (Clone PPT3) and culled 10 minutes later. Cryopreserved lymphocytes from TBLN, BAL, and PBMC were stained with Near-Infrared Fixable LIVE/DEAD stain (Invitrogen) and Rat Anti-Mouse IgG1-PeCy7 (Clone: RMG1-1, BioLegend) which labels the circulating cells and Mouse Anti-Pig CD3-Alexa Fluor 647 (Clone: BB23-8E6-8C8, BD Biosciences). As not all CD3 sites would be saturated by the i.v. anti-CD3 mAb, circulating cells are double labelled, whereas TRM cells are positive only for the IgG1-PeC7. Aurora Spectral Cytometer (Cytek Biosciences, USA) was used to analyse the data. Labelling of blood, TBLN, and BAL is shown in Supplementary Fig. S4.

### Gas chromatography/mass spectrometry

Deuterium enrichments in the DNA of sorted cell subpopulations were measured by gas chromatography/mass spectrometry (GC–MS) of the pentafluorobenzyl derivative as previously described [24, 25] monitoring the M + 1/M + 0 isotopomer ratio (m/z: 436/435) using negative chemical ionization (NCI) (GC2030/MS-QP2020NX, Shimadzu UK Limited, UK) with at least three replicate measurements for each sample. In pigs P1–P3 and P101–P104, plasma deuterium enrichments were also measured to assess body water enrichment, using the acetone method [26], monitoring ions m/z 57/58 in NCI mode (DB-17 column; Agilent 7890A/5975C, Agilent, CA, USA). Isotope ratios for deoxynucleotides and ^2^H_2_O were calibrated against standards of known isotopic enrichments and results were expressed as ‘DNA labelling (%)’ calculated as atoms percent excess (APE) [27].

### Statistics

Statistics were calculated in R v4.3.0; packages used include readxl (v1.4.2) and debug (v1.3.189) and GraphPad Prism v10 (GraphPad Software, Boston, MA). All *P* values reported are two-tailed.

## Results

### Labelling rates of porcine circulating lymphocyte subsets

Dosing at the levels used in Experiment 1 successfully achieved the body water enrichment levels required for measurable cellular labelling, ~1% in P1 and ~0.5% in P2/3 (Supplementary Fig. S1), approaching levels achieved in analogous human studies [28]. This corresponds to a body water turnover rate of about 2.5 l/day (the label administration rate divided by the enrichment rate). We attributed the post-peak decline in water labelling in P1 and the lower labelling rates in P2/3 to the rapid growth of these animals, reducing the effective dose per kg (Supplementary Fig. S1).

**Figure 3.**
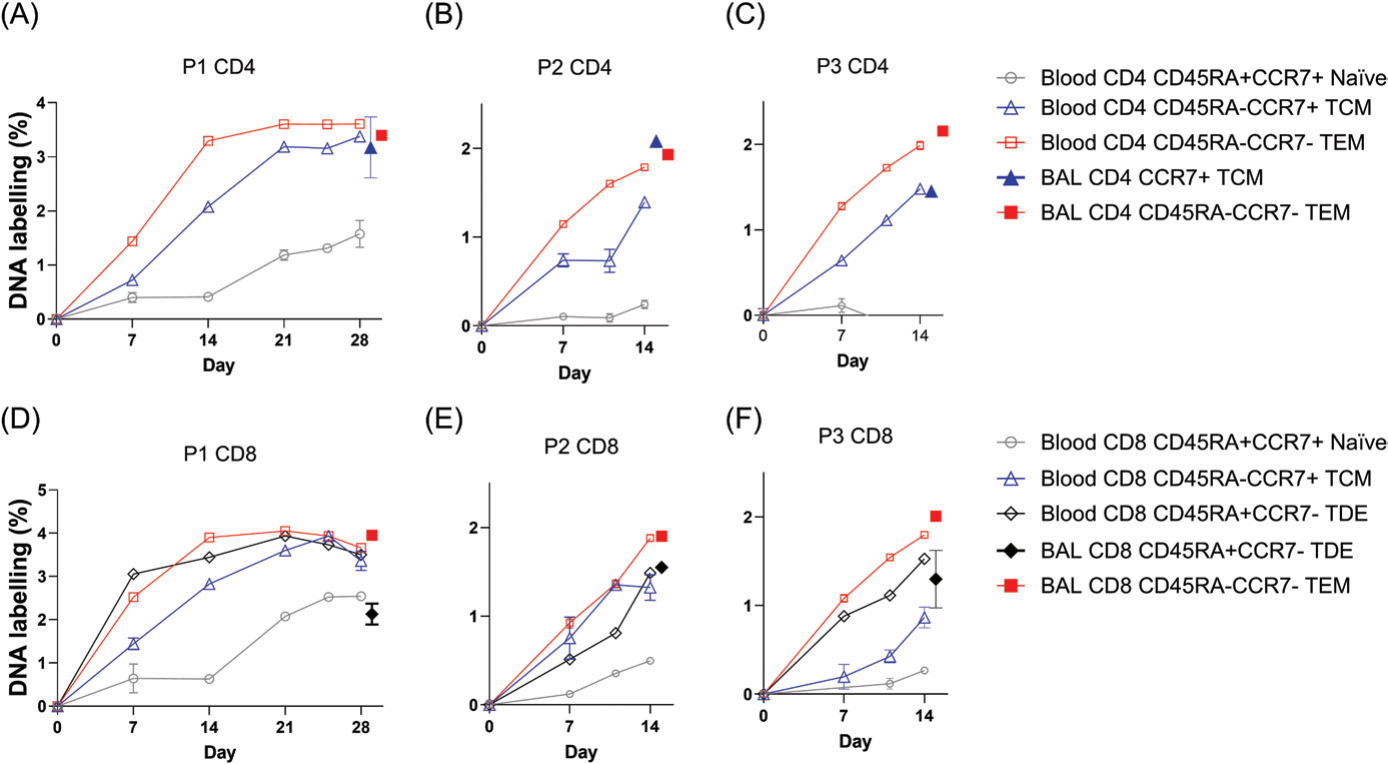
DNA deuterium labelling in blood over time compared with BAL labelling at the time of cull. DNA labelling (as APE) in sorted cell populations in blood (open symbols) and BAL (filled symbols) in pigs in Experiment 1. Upper panels (a–c) are CD4; lower panels (d–f) are CD8. Pigs were labelled for 28 days—pig 1, P1 (a and d); and 14 days—pig 2, P2 (b and e), and pig 3, P3 (c and f). Cell populations shown are CD45RA^+^CCR7^+^ naïve T cells (grey circles); CD45RA^−^CCR7^+^ central memory (TCM, blue triangles); CD45RA^−^CCR7^−^ effector memory (TEM, red squares); and for CD8^+^ cells, CD45RA^+^CCR7^−^ terminally differentiated effectors (TDE, black diamonds). BAL cells were taken on the day of culling (28, P1 or 14 days, P2/3) but are shown as filled symbols (same colour and shape as corresponding blood cells) displaced to the right (+1 day) for visualization alongside concurrently sampled blood cells. Error bars are standard deviations of analytic replicates—where not visible, error bars are less than symbol size.

T-cell subsets were separated by cell sorting into CD4 and CD8 naïve (TN, CD45RA^+^CCR7^+^), central memory (TCM, CD45RA^−^CCR7^+^), effector memory (TEM, CD45RA^−^CCR7^−^), and terminally differentiated effectors (TDE, CD45RA^+^CCR7^−^) (Supplementary Fig. S2) [23]. Blood cells labelled rapidly (Fig. 3) in a clear hierarchy TEM > TCM > TN for all three pigs for both CD4 (Fig. 3a–c) and CD8 (Fig. 3d–f) T-cell subsets. CD8 TDE in blood had labelling rates intermediate between TCM and TEM cells; CD4 TDE was too sparse for separation and analysis. By Day 21 in P1, DNA enrichment levels in memory CD4 and CD8 populations had plateaued suggesting that maximal labelling had been reached. This matches the predicted maximum labelling of a population of cells dividing in 1% heavy water which would be about 3.5–5.0% [28]. The implication is that by Day 21 (when the curves begin to flatten off), most memory T cells have been completely replaced by cells synthesized during the labelling period. These rates are substantially greater than those estimated from studies in adult humans and mice, probably because memory cell populations are still being built up in these young animals [29, 30]. The rate of division in the naïve population is also substantially greater than that seen in adult humans—most likely because the pig thymus reaches its maximum size between 12 and 24 weeks of age and that there is therefore still active thymopoiesis during the course of our experiments [29].

### High levels of labelling in memory T cells in BAL

**Figure 4.**
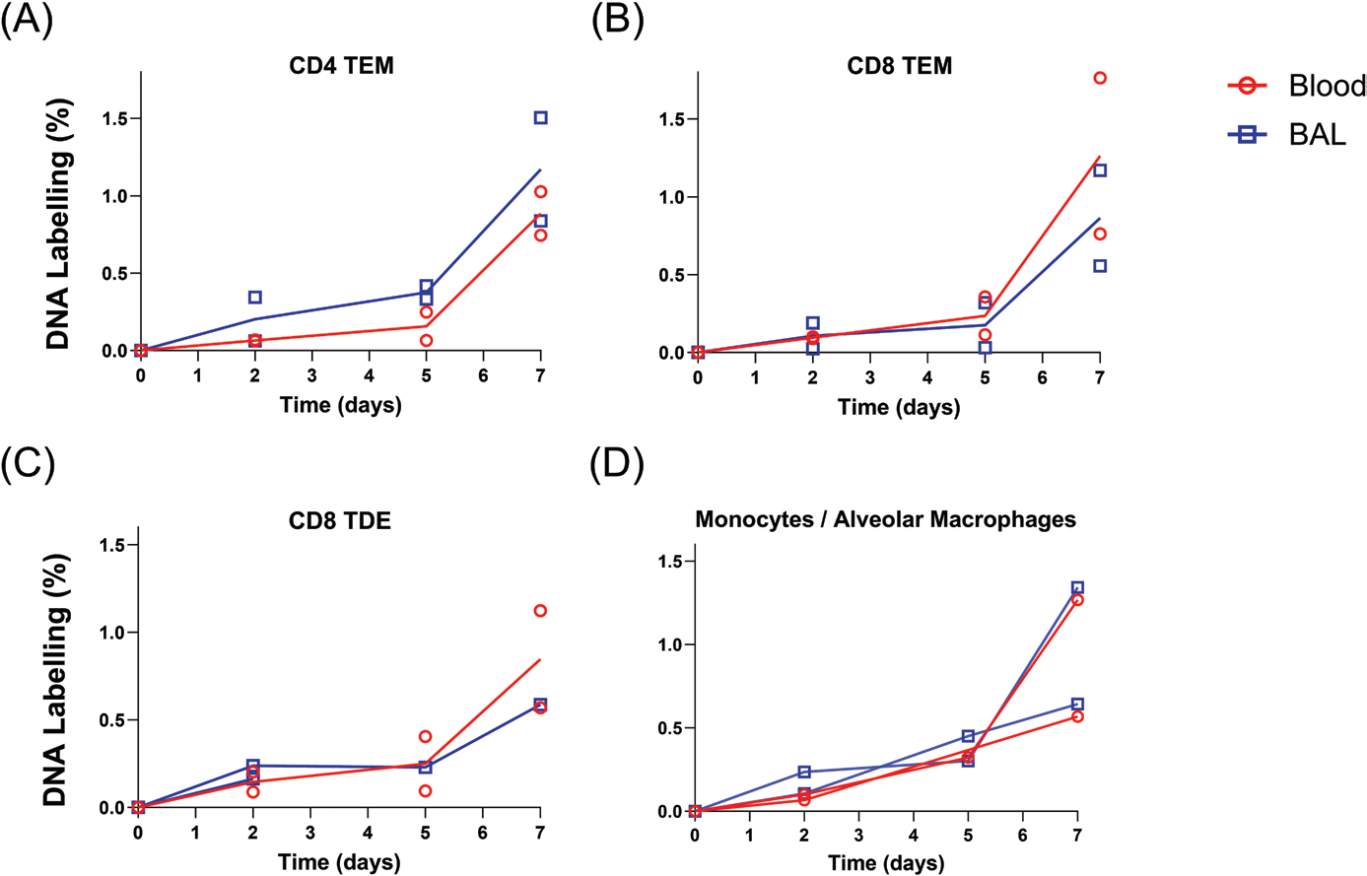
Time course of labelling in BAL and blood immune cells—Experiment 2. Graphs show labelling rates of cellular DNA in BAL (blue squares) and blood (red circles) memory cells after 2, 5, and 7 days of labelling in pigs P69/70, P71/72, and P75/76, respectively. Symbols show data from individual animals and lines connect means. Cells plotted are CD4 TEM (a), CD8 TEM (b), CD8 TDE (c), and blood monocytes/alveolar macrophages (d). Abbreviations as for Figure 2.

We next investigated the labelling of BAL TRM cells, defined elsewhere and here as T cells that are not labelled by short term intravenous CD3 antibody (Supplementary Fig. S4) [15, 21, 22]. As most of the T cells in BAL have a memory/activated phenotype we did not attempt to sort naïve cells [sorting CD4 TCM and TEM, and CD8 TEM and TDE (Supplementary Fig. S2)]. After 14–28 days of labelling (pigs P1–P3; Fig. 3) we found labelling rates in memory T cells in BAL very similar to those of their corresponding phenotype in blood—closed versus open symbols in Fig. 2. Although P3 was immunized with S-FLU vaccine by aerosol on the first day of heavy water labelling, the data showed very similar labelling rates to P1 and P2 and are included in subsequent analyses without reference to their vaccination state. At earlier time points, we also found very similar profiles, such that BAL label enrichment tracked their counterparts in blood throughout the time course (P69–72, P75, P76; Fig. 4a–c). Monocytes and alveolar macrophages, included for comparison, also showed very similar enrichment rates between BAL and blood (Fig. 4d). In some, but not all, animals we were also able to sort cells from lymph nodes. These data had more variance due to limited cell retrieval, but BAL and LN enrichments were also broadly similar at these later time points (Supplementary Fig. S5) as were lymph node and blood CD8^+^ and CD4^+^ memory cells (Supplementary Fig. S6).

### Labelling in BAL memory T cells lags behind blood T cells

**Figure 5.**
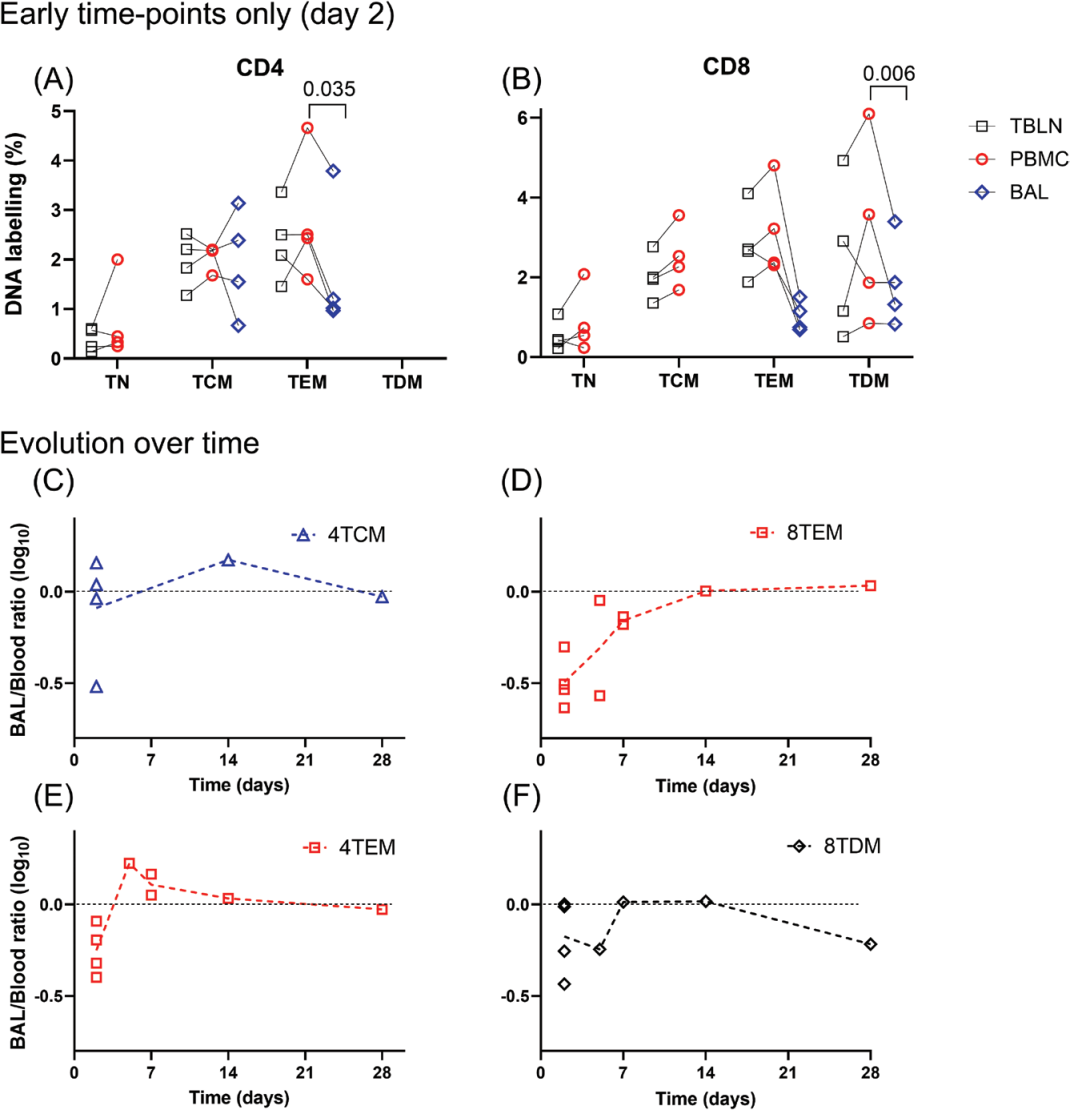
Rates of incorporation of deuterium in memory T-cell DNA by tissue origin, phenotype, and time. Upper frames: graphs show the distribution of labelling in memory T-cell subsets at the earliest time point measured (after 2 days labelling). Data were derived from pigs P101 to P104 (Experiment 3) and are shown separately for CD4^+^ (a) and CD8^+^ (b) memory T cells. *Y*-axis is DNA deuterium enrichment (normalized for body water enrichment) and *x*-axis is T-cell subset—abbreviations as for Figure 2. Black squares are lymph nodes; red circles are blood; and blue diamonds are BAL. *P* values shown are from paired *t*-tests comparing blood and BAL data after log transformation. Statistical comparisons between other tissues and between cell subsets (e.g. Blood TN versus Blood TCM) were not performed and are not shown. Lower frames: Distribution between BAL and blood of labelled cells shown as a time course of labelling ratio (expressed as log_10_) by cell subpopulation for CD4^+^ (c,e) and CD8^+^ (d,f) memory T cells; blue triangle, TCM; red square, TEM; black diamond, TDE. The line of parity (a BAL/blood ratio of one denoting equal labelling in both compartments), is shown on the log axis as a horizontal dotted line at zero.

**Figure 6.**
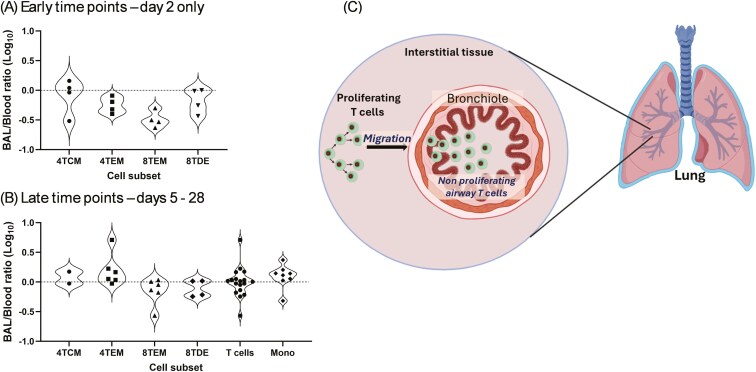
Comparison of labelling in BAL versus blood for memory T-cell subsets and model for porcine lung TRM maintenance most consistent with the data and previous literature. Comparison of labelling rates in parallel samples of memory T cells from the same labelled animal. Data are shown for 34 paired corresponding T-cell BAL/blood subsets (the same subset in the same animal at the same time) and 8 monocyte/AM paired subsets. Figures are violin plots of values expressed as log_10_ of the ratio of deuterium enrichment in DNA in BAL versus DNA enrichment in blood. Equal labelling in BAL and blood (a BAL/blood ratio of one) is shown on the log plot as a dotted line at zero. Data are shown separately for Day 2 only (a, Experiment 3, *n* = 16) when cells have been exposed to label for just 48 hours, and for cells exposed for 5 or more days of label (b, Experiments 1 and 2, *n* = 18). Cell subsets are TCM (circles), TEM (squares), and TDE (diamonds). Cell subset abbreviations: 4 and 8 prefix indicates CD4^+^ or CD8^+^, respectively; mono, monocytes/alveolar macrophages. (c) The proliferation of lung TRM occurs in lung interstitial tissue and labelled cells migrate to the bronchial epithelium and the airways. Dots in cell nuclei represent deuterium-labelled DNA in recently divided cells—at high proportions for illustrative purposes. For simplicity death *in situ* or efflux of cells from the airway are not shown.

In order to try to discriminate *in situ* proliferation of TRM from migration into the lung of cells that had proliferated elsewhere, we examined the Day 2 data from P69 and P70. However, absolute labelling rates in these animals were very low after such a short labelling period, close to the lower limit of mass spectrometric analysis, resulting in high coefficients of variation in the data. We therefore repeated the 2-day experiments with a larger prime and larger daily dose (P101–P104) to label dividing cells at higher deuterium enrichment levels. In these animals, we found similar labelling rates in TBLN and blood, as before. However, when we considered BAL memory T cells, we observed significantly lower labelling in BAL TEM than blood for both CD4 and CD8 memory cells (*P* = 0.035 and 0.006, respectively), suggesting a lag in the entry of labelled cells into these compartments (Fig. 5a and b). When we compared BAL/blood ratios—which control for variance caused by individual animal and labelling factors and differences between labelling protocols in different experiments—we observed a trend whereby the BAL/blood ratio in effector memory cells began below one (represented by zero on the log scale) at Day 2 but rose thereafter to approach unity after 5–14 days (Fig. 5d and e); in TCM and TDM this effect was not clearly seen (Fig. 5c and f). Strikingly, whereas in samples retrieved at or after Day 5, the ratio clustered around unity (median: 1.02; IQR: 0.71–1.21, *n* = 18), suggesting equilibration or equivalence, ratios at Day 2 were lower (median: 0.53; IQR: 0.31–0.97; *n* = 16), especially in CD8 TEM (Fig. 6).

## Discussion

In humans, the majority of circulating T memory cells have been shown to divide more rapidly than naïve T cells, perhaps representing a mechanism for limiting the survival of memory clones in the absence of further exposure to antigen [3, 31, 32]. In addition, a numerically smaller fraction may persist as non-dividing memory stem cells [5, 6]. However, although it is clear that antigen-specific populations of memory TRM can persist in non-lymphoid tissues for long periods of time without replenishment from the blood, there is much less information indicating whether TRM populations persist by longevity of individual cells or are maintained by ongoing cell division, as for circulating memory T cells [8]. To resolve this uncertainty, we directly compared the proliferation rates of TRM in a specific relevant setting, porcine lung airways, with circulating T cells during *in vivo* stable isotope tracer studies.

We first developed a novel heavy water labelling protocol for porcine leucocytes for young pigs. Heavy water labelling has previously been successfully used in goats given 4%-labelled drinking water *ad libitum* for 4 weeks, to study memory cell dynamics in bone marrow [33]. We found that oral labelling could be readily achieved in pigs using daily doses of flavoured heavy water and attained levels of labelling in plasma and cells, which were in the readily measurable range. In our initial 28-day study, we found that most circulating memory cells were fully labelled by the end of the labelling period, so added a priming dose to achieve rapid body water labelling and progressively reduced the labelling time (Fig. 2), enabling the study of rapid events in cell proliferation and exchange.

Initially, we studied the proliferation rates of peripheral blood T-cell subsets and the results mirror the findings in humans [3]. Thus, peripheral blood porcine T cells considered to be phenotypically naive (CD4 and CD8 cells that are CD45RA^+^CCR7^+^) incorporated less label than either central memory cells (CD45RA^−^CCR7^+^) or more differentiated or activated subsets (CD45RA^-^CCR7^-^ effector memory or CD45RA^+^CCR7^−^ terminally differentiated effector memory). We also noted almost identical labelling rates in blood and lymph node samples in corresponding subsets, consistent with a rapid exchange between these two compartments. Although the difference between phenotypically naïve and memory subsets is clear (Fig. 3), porcine naïve cells labelled more rapidly than those in adult humans, almost certainly due to the efflux of recently divided thymocytes into the circulating naïve T-cell pool from a highly functional thymus in pigs that are only 6–10 weeks of age [29].

When we compared labelling rates in phenotypically defined T-cell subsets in blood and BAL at later time points (5–21 days) we noted strikingly similar labelling (Figs. 3 and 4). However, when we studied animals labelled for only 48 hours, we found a lower level of labelling in some BAL memory subsets. Four alternative mechanistic hypotheses might account for the persistence of BAL TRM (Fig. 1).

The first is that BAL is maintained by the longevity of individual TRM cells. Our data are not consistent with this model as this hypothesis would predict that BAL cells would remain unlabelled, which we do not see in this study. In the second, BAL TRM derives from the entry of recently proliferated cells into the lungs from blood; the lower labelling at Day 2 can then be explained by a delay in equilibration. However, the observation that by 5 days blood and BAL show identical labelling indicates that there would need to be a very considerable daily influx of cells (and presumably a balancing efflux) to account for our results. Although in previous experiments BAL TRM was contaminated by up to 9% of blood-derived T cells [15], three pigs infused with CD3 mab in parallel with the pigs labelled with heavy water showed <2% contamination (Supplementary Fig. S4). Such a low proportion of contaminating blood-derived T cells could not account for the rapid labelling of BAL TRM we observe. Furthermore, analysis of the murine TCR repertoire in lymphoid tissues and the lung indicates that these differ, suggesting that TRM does not rapidly equilibrate with T cells in the lymphoid system [34]. Similarly, although large CD8 T-cell clones are shared between blood, lymph nodes, and lung TRM in humans, more detailed analysis indicates that there are tissue-specific clonal expansions and that tissues maintain clonal diversity with age, whereas large age-associated clonal expansions are common in circulating blood memory subsets [35]. Our own data also indicates that the proportions of T cells recognizing three different peptide tetramers in influenza-immunized pigs differ between blood and BAL, again indicating a restriction on free (and rapid) exchange between these sites [23]. Overall, these data strongly indicate that the rapid influx of cells is not the explanation for the labelling of airway T cells that we observe.

A third hypothesis is that cells with similar (though not identical) phenotypes divide at similar rates in different locations, with substantial rates of cell division *in situ* among BAL memory cells, albeit slightly slower than in corresponding cells in the blood/LN compartment. Few studies have compared directly the division rates of circulating and tissue-resident cells in large animals using DNA labelling methods. However, although Ki67 staining may indicate a reduced rate of cell division of human TRM compared to blood T cells [13], a goat study identified a disparity between the results of *in vivo* labelling and Ki67 staining, casting some doubt on the use of Ki67 to measure cell proliferation *in vivo* [33]. Nevertheless, the short delay in labelling of BAL TRM, which we observe, could be compatible with a slower rate of *in situ* proliferation of airway TRM than circulating cells, as suggested by the Ki67 data [13]. However rapid proliferation of airway TRM (BAL TRM) is not compatible with the observation that when congenic CD45.1 cells are introduced into the airways of CD45.2 mice, there is rapid attrition of the congenic cells and replacement by host CD45.2 cells, suggesting that the airway population is relatively rapidly replaced by cells originating and dividing elsewhere [36].

We therefore favour a fourth hypothesis, that cell division is taking place in a different lung compartment, from whence cells migrate into the superficial layers of airway epithelium and are harvested as BAL (Fig. 6c). If BAL TRM are rapidly replaced from another source what is the origin of the cells? Such replacement was first thought to be from circulating TEM [37]. However, whereas a comparison of the TCR repertoire of airway and lung interstitial TRM indicates considerable overlap, there is little overlap between either the lung TRM population and the lymphoid system. Furthermore, murine BrdU labelling experiments indicate that lung interstitial TRM proliferate and give rise to non-dividing airway TRM, although the proliferation rate of interstitial TRM appeared lower than that of splenic memory cells. In addition, antigen-specific airway T cells could be maintained when circulating memory cells, but not lung interstitial TRM were depleted [11, 12]. This model, in which airway TRM are non-dividing *in situ* but are replenished from a lung interstitial TRM population with a similar TCR repertoire, dividing at a similar rate to circulating blood/LN cells, would be consistent with our data. It would explain satisfactorily the relatively high labelling rates at later time points and also the delay in BAL labelling seen at Day 2, due to the time interval required for migration from the lung parenchyma to the airway.

Comparison of our data with murine studies is complicated by the fact that the pigs are by no means similar to specific pathogen-free mice. Murine studies with ‘dirty’ pet shop mice indicate that in such animals the number of TRM continues to increase over time without increasing the rate of disappearance of previously induced antigen-specific populations [8]. Thus, in conventionally housed animals continually exposed to new antigens, the rate of entry of cells to non-lymphoid tissues is greater than in clean SPF inbred mice. In addition, analysis of numbers of TRM in human tissues of different ages indicates that these increase during the first few years of life [30]. The pigs used in our studies were only 6–7 weeks of age at the start of our experiments, so TRM populations would most likely be still increasing, but it seems unlikely that these gradual changes would greatly influence the results of short timescale labelling experiments.

Investigation of the kinetics of monocytes and alveolar macrophages was not the focus of this investigation; these cells were included as a ‘positive control’. However, the observed similar rapid acquisition of deuterium labelling in both blood and BAL compartments (Figs. 4d and 6b, and Supplementary Fig. S7) is consistent with the known characteristics of blood monocytes—that they are produced rapidly in bone marrow, have a short dwell time in blood, and rapidly exchange with tissues—although we cannot from this data alone exclude *in situ* proliferation. In humans, monocytes leave the blood within a day of entering the circulation from bone marrow [38].

In summary, our experiments (i) have established a methodology for studying cellular kinetics in a large animal with many similarities to humans, (ii) confirmed that circulating naïve cells divide more slowly than memory T cells, (iii) shown rapid equilibration between the blood and lymph node memory compartments (iv) indicated that airway TRM is actively replenished by cell division at a substantial rate. Although our data cannot experimentally distinguish between alternative mechanisms for TRM replenishment: the influx of recently divided cells from blood, *in situ* proliferation of airway TRM, or migration of recently divided cells from a dividing lung interstitial compartment (Fig. 6c); when taken together with other data, the latter appears most likely to explain the surprisingly high labelling rates seen in the airway TRM population in this highly relevant large animal model.

## Supplementary Material

kyaf007_suppl_Supplementary_Figures_S1-S7

## Data Availability

The raw data supporting the conclusions of this article will be made available by the authors, without undue reservation.
