# Supplementary material for: Proliferation makes a substantive contribution to the maintenance of airway resident memory T-cell subsets in young pigs
Source: Discov Immunol. 2025 Apr 12;4(1):kyaf007. doi: 10.1093/discim/kyaf007 (PMC12076203; doi:10.1093/discim/kyaf007)
Supplement: kyaf007_suppl_Supplementary_Figures_S1-S7 [file kyaf007_suppl_supplementary_figures_s1-s7.pdf]

## Supplementary Material

# Contents

Supplementary Figure S1. Weight trends and deuterium labelling of body water in animals P1-P3 .......................................................... 2

Supplementary Figure S2. Gating strategy for separation of memory cells from BAL, PBMC and Lymph node ....................................... 3

Supplementary Figure S3. Enrichment of CD3 cells following CD172 bead depletion..........................................................................4

Supplementary Figure S4. Porcine TRM cells.........................................................................................................................5

Supplementary Figure S5. Concurrent labelling of cells in Lymph node and BAL ........................................................................... 6

Supplementary Figure S6 Concurrent labelling of CD4+ T cells in Blood and LymphNode..........................................................7

Supplementary Figure S7 Supplementary Figure S7. Labelling in blood monocytes and BAL alveolar macrophages.........................8

### Supplementary Figure S1. Weight trends and deuterium labelling of body water in animals P1-P3

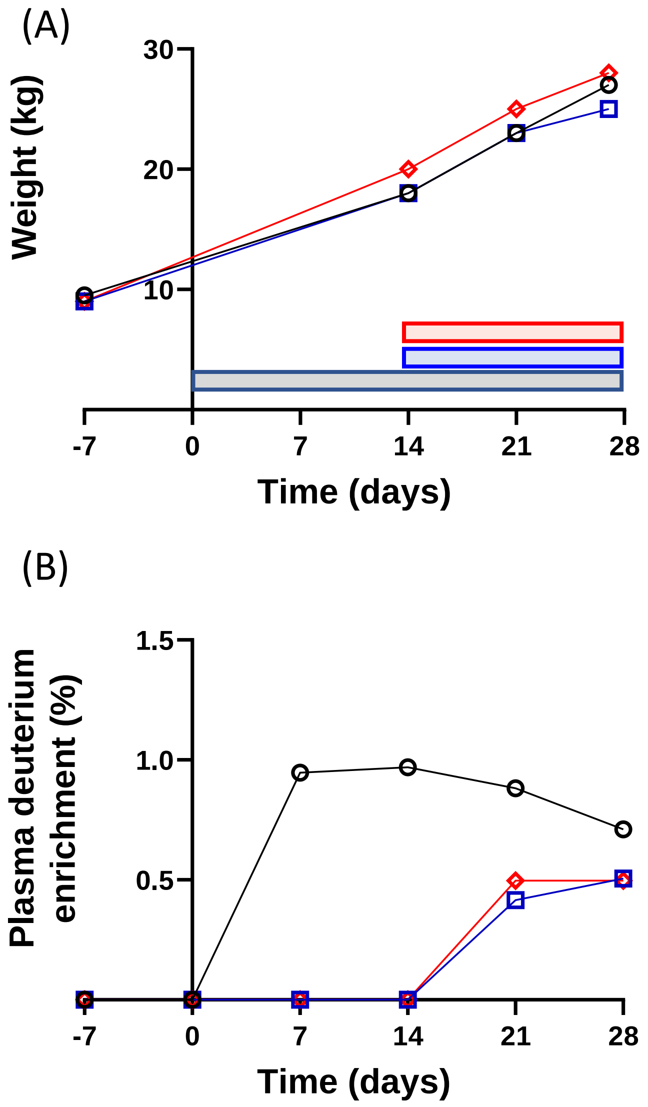


(A) Weight trends for pigs P1 (black lines/grey bar), P2 (blue), and P3 (red). Horizontal bars show labelling periods with culling at day 28.
(B) Plasma deuterium enrichments for P1 (black), P2 (blue) and P3 (red) at corresponding time-points expressed in atoms percent excess.

## Supplementary Figure S2. Gating strategy for separation of memory cells from BAL, PBMC and Lymph node

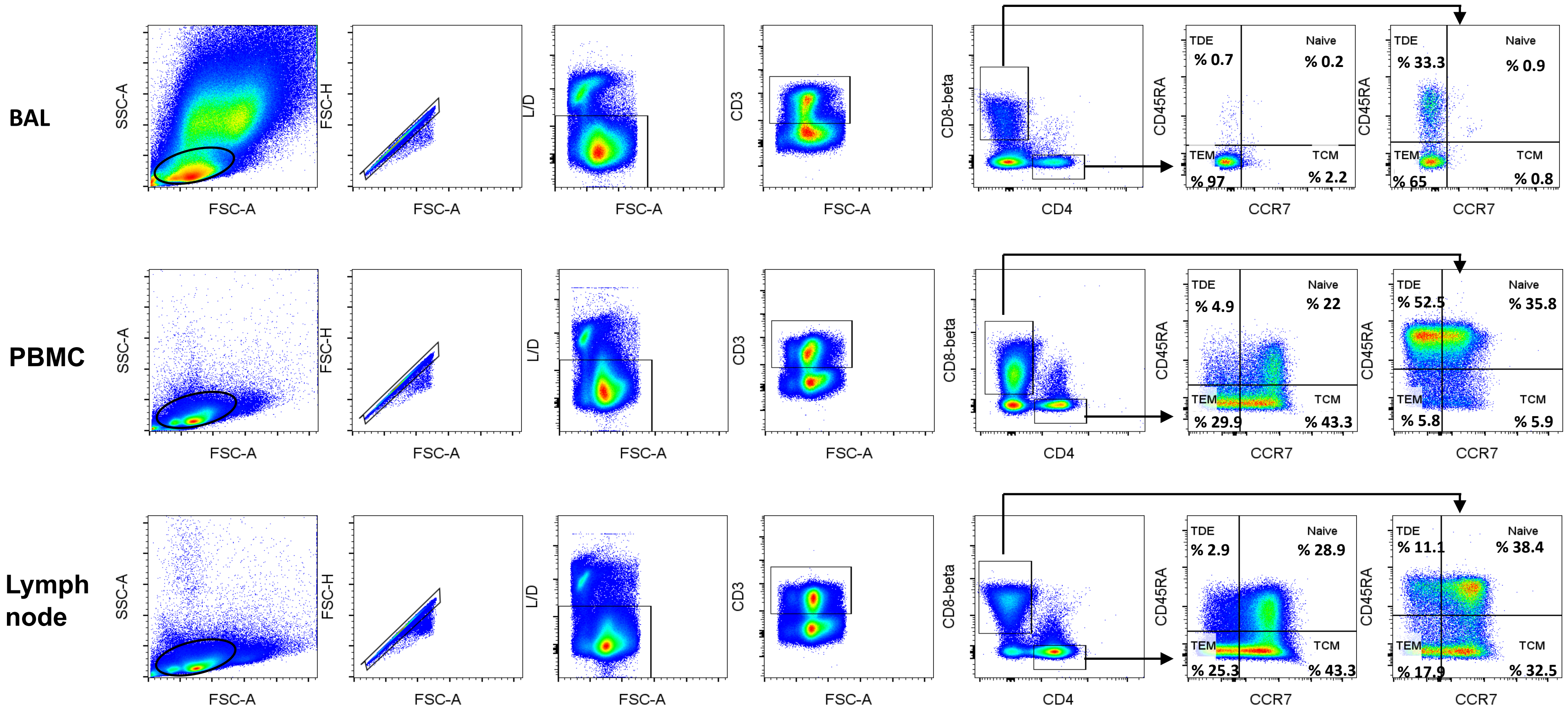

**Supplementary Figure S3. Enrichment of CD3 cells following CD172 bead depletion**

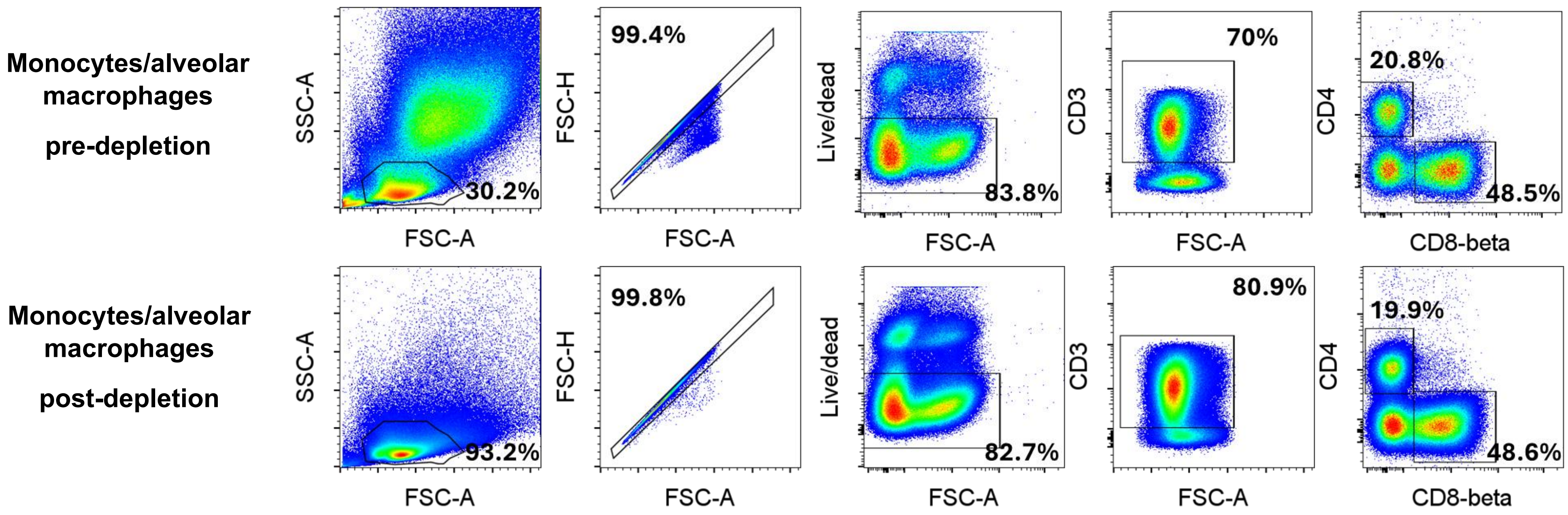

## Supplementary Figure S4. Porcine TRM cells

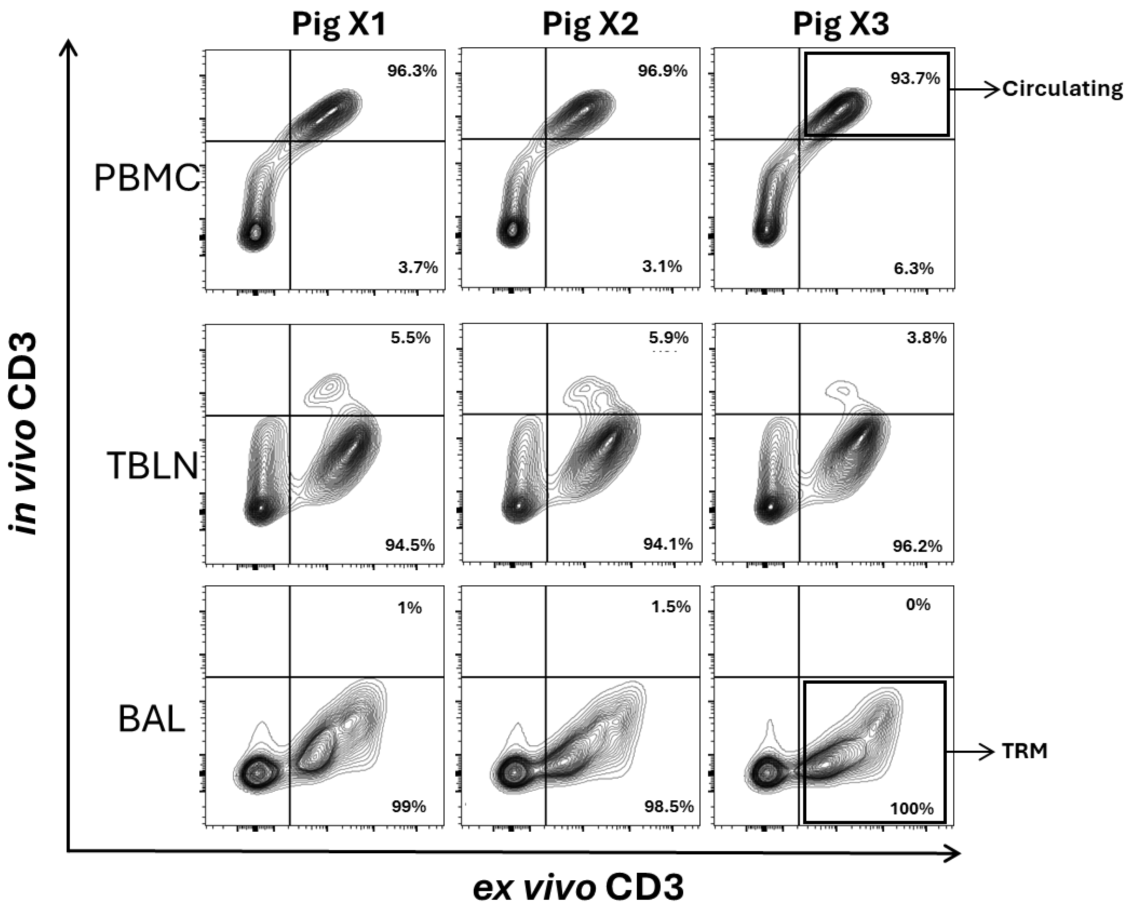


Three pigs were infused intravenously with CD3 Ab and sacrificed 10 min later. Lymphocytes were isolated and stained with anti-mouse IgG1-PeCy7, and CD3 Ab labelled with APC. As the infused CD3 does not saturate all CD3 sites, blood and TBLN T cells are double positive, while more than 98% of the BAL cells are inaccessible for CD3 representing TRM.

## Supplementary Figure S5. Concurrent labelling of cells in Lymph node and BAL

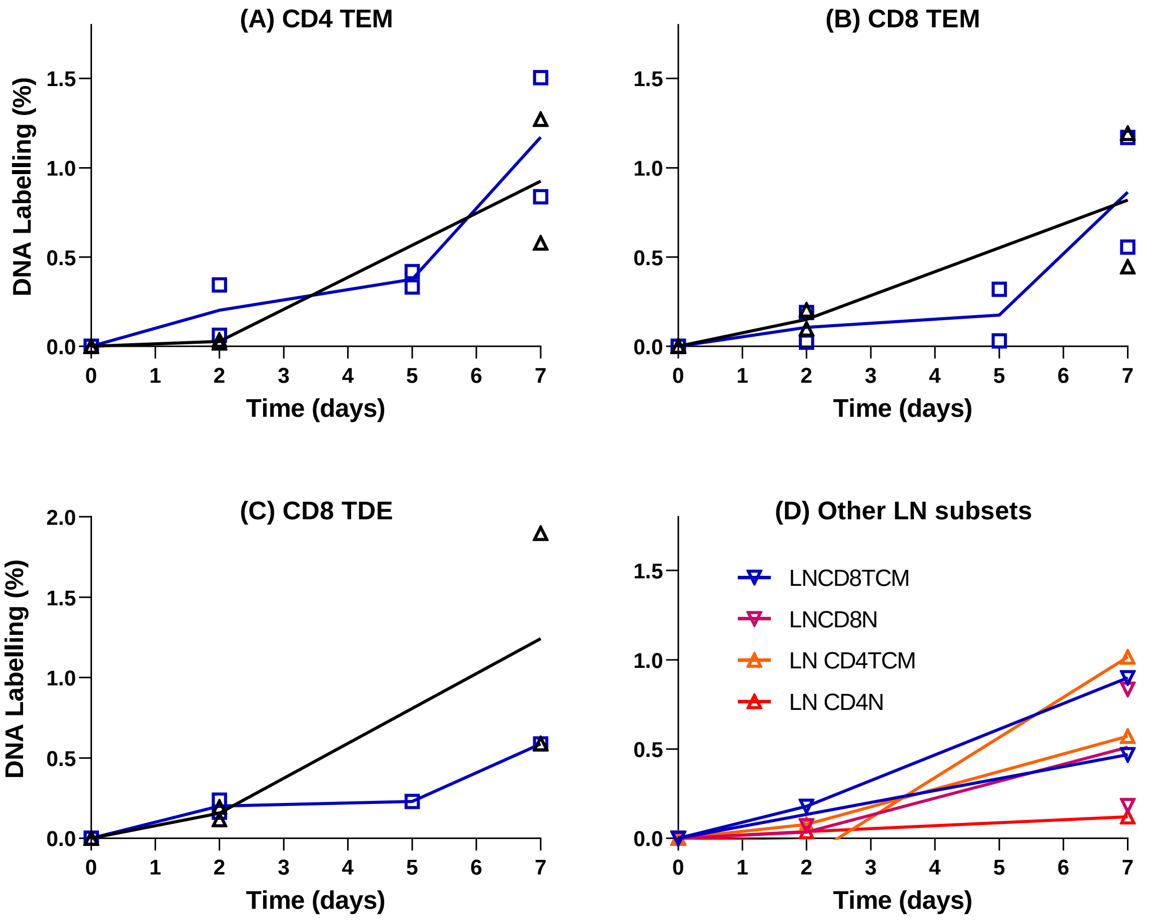


Labelling rates of cellular DNA in memory cells after 2, 5 and 7 days of labelling in pigs 69/70, 71/72 and 75/76 respectively. Symbols show paired data from BAL (blue) and lymph node cells (black) in individual animals where paired data were available for the same subset in the same animal; lines show means of n=2. Cell subsets are CD4 TEM (A), CD8 TEM (B), CD8 TDE (C). Frame (D) shows lymph node labelling curves for other subsets where paired BAL data was not available.

## Supplementary Figure S6. Concurrent labelling of CD4+ T cells in Blood and Lymph Node

### LN versus Blood - CD8

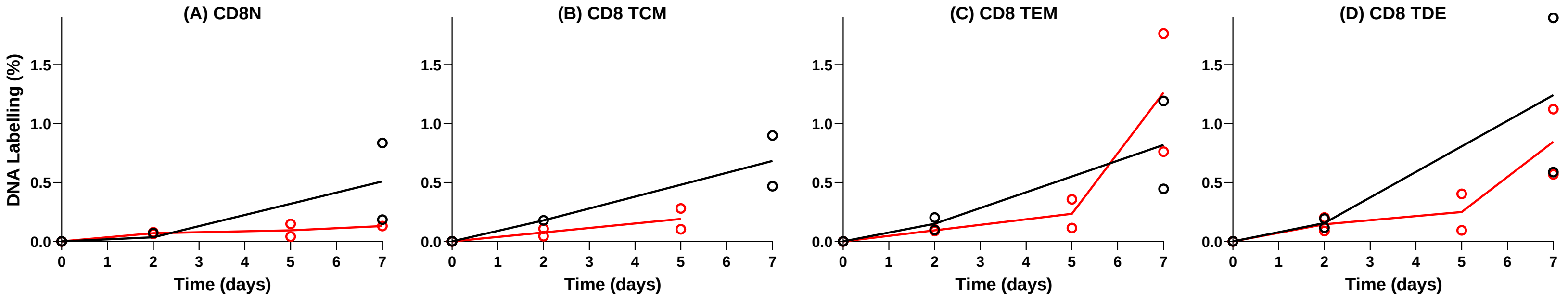


### LN versus Blood - CD4

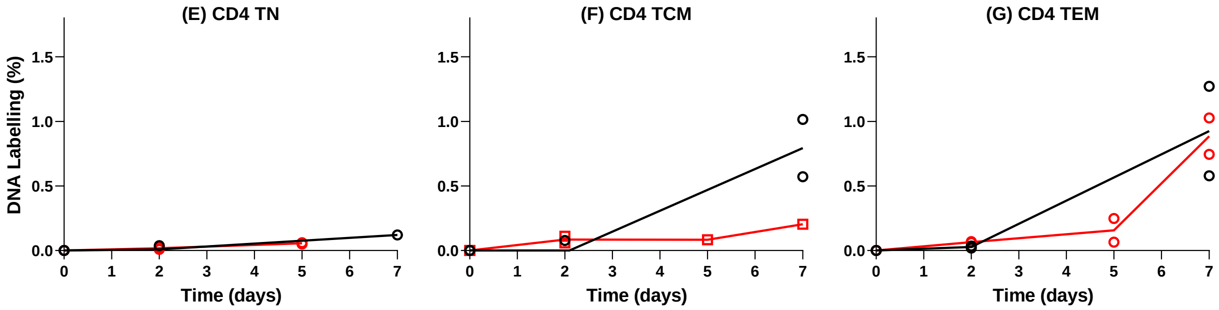


Labelling rates of cellular DNA in CD8+ and CD4+ memory cells after 2, 5 and 7 days of labelling in pigs 69/70, 71/72 and 75/76. Symbols show data from individual animals and lines show means of n=2. Blood cells are shown in red and corresponding lymph node cells in black. Cells plotted are CD8N (A), CD8 TCM (B), CD8 TEM (C), CD8 TDE (D), CD4 TN (E), CD4 TCM (F), and CD4 TEM (G).

**Supplementary Figure S7. Labelling in blood monocytes and BAL alveolar macrophages**

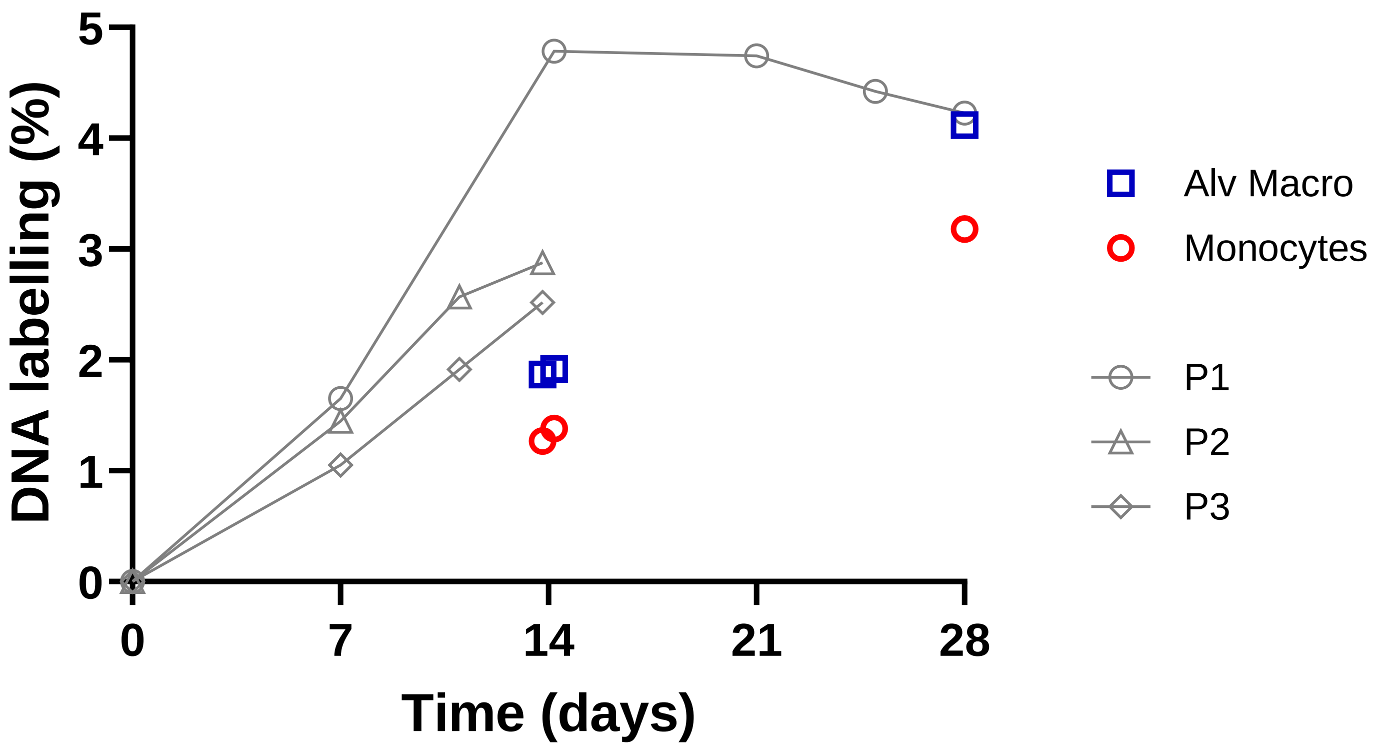


DNA labelling in sorted blood monocytes (red circles) and alveolar macrophages (blue squares) from animals P1-P3. P1 was labelled for 28 days; P2 and P3 for 14 days. Data for day 14 are shown slightly displaced by time for clarity. Grey symbols show contemporaneous labelling in granulocytes from P1 (circles), P2 (triangles) and P3 (diamonds).
